# Research progress on the relationship between long QT syndrome (LQTS) and epilepsy: A review

**DOI:** 10.1097/MD.0000000000048282

**Published:** 2026-04-10

**Authors:** Qing Li, Ling-Bing Meng

**Affiliations:** a Department of Nutrition, The First Affiliated Hospital (Southwest Hospital) of Army Medical University, Chongqing, China; b Cardiometabolic Medicine Center, National Clinical Research Center for Cardiovascular Diseases, Fuwai Hospital, National Center for Cardiovascular Diseases, Chinese Academy of Medical Sciences and Peking Union Medical College, Beijing, China; c Department of Cardiology, National Clinical Research Center for Cardiovascular Diseases, Fuwai Hospital, National Center for Cardiovascular Diseases, Chinese Academy of Medical Sciences and Peking Union Medical College, Beijing, China; d State Key Laboratory of Cardiovascular Disease, Beijing, China; e Translational Medical Center, Weifang Second People’s Hospital, Weifang, China.

**Keywords:** epilepsy, ion channels, long QT syndrome (LQTS), nervous system, pathogenic mechanisms

## Abstract

Long QT syndrome (LQTS) is a hereditary disorder caused by cardiac electrophysiological abnormalities, while epilepsy is a group of neurological disorders characterized by brain electrical abnormalities. Despite involving different physiological systems, recent research suggests a potential interconnection between LQTS and epilepsy. In this review of previous studies, we summarize independent investigations of LQTS and epilepsy, emphasizing efforts to identify common mechanisms between these 2 diseases. By analyzing biological mechanisms such as ion channel dysfunction and neuronal excitability, we propose some shared underlying pathophysiological features between LQTS and epilepsy. We further discuss clinical manifestations and diagnostic challenges, focusing on similar symptoms that patients may exhibit and how to differentiate between these 2 conditions in clinical practice. Finally, we provide insights into future research directions, highlighting the potential benefits of advancements in this field for clinical practice and treatment strategies. Through in-depth exploration of the relationship between LQTS and epilepsy, we aim to contribute to more precise approaches for the prevention and treatment of these 2 diseases.

## 1. Introduction

Long QT syndrome (LQTS) is a common inherited cardiac disorder characterized by abnormal cardiac repolarization leading to prolonged QT intervals, increasing the risk of arrhythmias and sudden cardiac arrest. This syndrome is primarily caused by genetic mutations involving various ion channel proteins related to cardiac electrophysiology.^[1,2]^ Clinical manifestations of LQTS include syncope, palpitations, and, in severe cases, may lead to sudden cardiac arrest.^[3,4]^ Clinically, LQTS is classified into several subtypes, with LQT1, LQT2, and LQT3 being the most common subtypes.^[5]^ LQT2, caused by mutations in the HERG gene, results in diminished delayed rectifier potassium ion channel current (IKr). It is characterized by prolonged QT intervals on electrocardiograms and the occurrence of Torsades de Pointes, a type of polymorphic ventricular tachycardia, leading to episodes of seizure-like syncope and sudden death.^[6]^ Due to these clinical presentations,^[7]^ LQTS patients are often misdiagnosed as having epilepsy.^[8]^

Epilepsy is a group of chronic neurological disorders caused by abnormal electrical activity in the brain, resulting in episodic disruptions of brain function known as seizures.^[9,10]^ Seizure manifestations vary widely and can include brief loss of consciousness, convulsions, and sensory abnormalities. Epilepsy may arise from genetic mutations, brain injury, or other neurological disorders.^[11–13]^ In addition to seizures secondary to acquired epilepsy, in recent years, some cases of primary and cryptogenic epilepsy have been linked to ion channel dysfunction.^[14,15]^ Similarly, LQTS is a primary cardiac ion channel disorder caused by abnormal ion channel function, with both conditions closely associated with ion channels such as sodium and calcium ions.^[16–19]^

Numerous studies have indicated clinical similarities between epilepsy and LQTS, and there have been several reported cases suggesting the coexistence of these 2 conditions, leading to potential misdiagnosis.^[20–23]^ Despite being diseases involving the heart and nervous system, respectively, research has proposed similarities in the pathophysiological mechanisms of epilepsy and LQTS, making accurate diagnosis challenging.^[24]^ Both conditions can present with recurrent syncope, loss of consciousness, seizures, sudden death, and similar family histories, imposing significant psychological stress and life-threatening risks on patients and their families. Therefore, the importance of correctly identifying and providing standardized management for these conditions cannot be overstated. In-depth research into their relationship is crucial for a comprehensive understanding of the pathogenesis of these 2 diseases and for developing more effective treatment and management strategies for patients.

## 2. Understanding and development of LQTS and epilepsy

The 1st description of LQTS can be traced back to the late 1950s and early 1960s. Currently, scholars in the field generally agree that the fundamental cause of LQTS is genetic mutations associated with potassium and sodium ion channels in the heart.^[25,26]^ These gene mutations lead to dysfunction in ion channel activity, causing abnormal myocardial repolarization during the action potential, ultimately resulting in prolonged QT intervals and triggering arrhythmias.^[19,27,28]^ This perspective integrates research findings on the pathogenesis of LQTS, emphasizing the crucial impact of genetic abnormalities on cardiac electrophysiology.

LQTS episodes are often triggered by physiological or emotional factors such as exercise, shock, or excitement. Patients may experience sudden fainting during physical activity or excitement, accompanied by brief loss of consciousness.^[29–31]^ They may also feel a rapid or irregular heartbeat due to arrhythmias, and these life-threatening arrhythmias can potentially lead to sudden cardiac arrest.^[17,32,33]^ Inherited arrhythmias are a major cause of unexplained sudden death, accounting for 20% to 25% of cases.^[34]^ The severity of symptoms is related to the duration of arrhythmias and the ability to spontaneously revert to sinus rhythm or receive timely electrical defibrillation. Congenital LQTS is more common in children and adolescents, with an average onset age of 14 years, similar to the age of onset for epilepsy syndromes.^[35]^

In the past, it was believed that the epileptic loss of consciousness or seizures in LQTS patients was due to brain ischemia and hypoxia caused by arrhythmias.^[36–39]^ However, there are significant differences in seizure characteristics or electroencephalogram (EEG) discharges among different types of LQTS patients, indicating that various factors contribute to distinctive seizures in different LQTS subtypes.^[40,41]^ Among the 17 identified subtypes of LQTS, the highest frequency of reported epilepsy symptoms and similar manifestations is observed in LQTS2 patients.^[38,42]^ Primary epilepsy and LQTS share, to some extent, similar pathogenic mechanisms, as both belong to ion channel disorders affecting the brain and heart, respectively.^[43]^

Mutations in the human ether-a-go-go-related gene (hERG) (KCNH2) gene can lead to the loss of hERG channel function, resulting in the occurrence of LQT2.^[44,45]^ Increased hERG channel function, on the other hand, causes the development of short QT syndrome type 1. The hERG gene encodes the α subunit of the rapidly activating delayed rectifier potassium current (IKr) channel.^[46,47]^ Mutations in the hERG gene can lead to IKr channel inactivation, reduced or absent outward potassium current, prolonged repolarization of myocardial cells, and increased susceptibility to ventricular arrhythmias, particularly Torsades de Pointes ventricular tachycardia, and even ventricular fibrillation.^[48–50]^ This subunit is expressed in various tissues, including the heart and brain, causing abnormal repolarization in the heart and an epileptic phenotype. The hERG channel encoded by the KCNH2 gene plays a crucial role in neural signal transmission, especially in active hippocampal astrocytes.^[51]^ Abnormal function of this channel may disrupt normal potassium ion concentrations inside and outside brain cells, increasing susceptibility to recurrent epileptic activity. This situation aligns with the symptoms of increased epilepsy susceptibility observed in LQT2 patients.^[42,52,53]^ Therefore, the abnormal function of the KCNH2 channel may be a contributing factor to the increased occurrence of epilepsy in LQT2 patients.

Miyazaki et al^[54]^ identified 5 out of 6 perinatal LQTS patients (83%) among 21 infantile LQTS cases who were diagnosed with epilepsy, with 4 of them (67%) exhibiting developmental disorders. The total developmental quotient for the 5 perinatal LQTS patients with epilepsy ranged from 17 to 72 (median 67). Among 8 perinatal LQTS patients with neurological disorders, 3 of them had previously reported cases, experiencing seizures between 2 days and 2.5 years of age, with 5 exhibiting developmental disorders. Mutations in these 8 patients were located in the transmembrane helix, D3/S4–S5 linker, D4/S4 regions of KCNH2, or D4/S6 segment of SCN5A. Zhou et al^[23]^ investigated a 25-year-old female with a family history of epilepsy and LQTS, who had experienced seizures since the age of 7, and subsequently had postpartum seizures at 18 and 25 years of age. Genetic testing revealed a heterozygous variant in the KCNH2 gene [c.2230 (exon 9) C > T. Arg744Ter, 416, NM_000238, rs189014161] found in both the proband and her mother. Additionally, numerous similar cases have been reported.^[6,38,55,56]^ LQTS patients show a significantly higher proportion of abnormal EEGs (including slow waves and seizure-like discharges in the range of 2–7 Hz) compared to healthy controls.^[54]^ Approximately half of LQT2 patients (36/77 cases, 46.8%) exhibit an epileptic phenotype, which is markedly higher than in other LQTS subtypes.^[57]^ Similarly, LQT2 patients have a higher personal history or previous diagnosis related to epilepsy compared to other LQTS subtypes.^[58]^ Therefore, it is speculated that there may be a certain correlation between LQTS and epilepsy. Epileptic seizures can also affect cardiac function, leading to arrhythmias and blood pressure abnormalities. Studies have found that the most common arrhythmia after epileptic seizures is ventricular arrhythmia, and abnormalities in cardiac channels may play a crucial role in causing sudden death related to epilepsy.^[59,60]^ Thus, there is a growing perspective that epileptic seizures in LQTS patients may not only be due to cerebral neuron ischemia and hypoxia but may also involve disturbances in potassium and sodium ion channels within neurons, leading to blocked neuronal repolarization.^[41,61,62]^ Additionally, astrocytes may disrupt the clearance of potassium ions around neurons, causing abnormal changes in potassium ion concentrations inside and outside neurons, resulting in abnormal neuronal discharge.^[63–66]^ These abnormal changes may be the result of the mutual influence between epileptic seizures and arrhythmias.

## 3. Mechanisms of onset for LQTS and epilepsy

### 3.1. Mechanisms of LQTS onset

LQTS is a hereditary arrhythmia disorder caused by abnormalities in cardiac ion channel function.^[67]^ The pathogenic mechanism of LQTS primarily involves abnormalities in the depolarization and repolarization processes of myocardial cells, leading to the prolongation of action potentials in ventricular muscle cells, manifested as prolonged QT intervals.^[55]^ Most LQTS patients carry mutations in specific genes encoding cardiac ion channel proteins located on the membrane of myocardial cells, regulating cardiac action potentials.^[68,69]^ The most common genes associated with LQTS are KCNQ1, KCNH2, and SCN5A, encoding the IKs, IKr, and INa channel proteins, respectively, corresponding to the pathogenic genes for LQTS1, LQTS2, and LQTS3 subtypes.^[70–72]^

The pathogenic gene for LQT1, KCNQ1, encodes the alpha subunit of the slow-activating delayed rectifier potassium current (IKs) channel, known as the Kv7.1 channel. This channel consists of 4 alpha subunits, each comprising 6 transmembrane segments (S1–S6), 1 P-loop with ion selectivity function (the extracellular side connecting regions between S5 and S6), and amino and carboxyl terminals located internally in the cell.^[73]^ The P-loops of the 4 subunits form the selective channel pore of the Kv7.1 channel. The amino terminal contains approximately 100 amino acids, including multiple phosphorylation sites for protein kinase A, while the carboxyl terminal has a conserved amino acid sequence.^[74,75]^ The Kv7.1 channel lacks inactivation characteristics and only exhibits voltage-gated properties.^[76]^ Approximately 250 KCNQ1 mutations have been reported to cause LQT1, commonly characterized by varying degrees of reduction and/or dysfunction of Kv7.1 channels, or abnormal cellular membrane localization.^[77,78]^ This ultimately results in reduced IKs, causing delayed repolarization, prolonged action potential duration and QT intervals, making it prone to early afterdepolarizations (EAD) and triggering malignant arrhythmias.^[79–81]^

Mutations in the SCN5A gene, which encodes the alpha subunit of the cardiac voltage-gated sodium ion channel (Nav1.5), lead to LQT3.^[79,80]^ The Nav1.5 channel comprises 4 homologous domains (DI–DIV), with amino acid sequences connecting DI–DIV located internally in the cell. Each domain consists of 6 transmembrane segments (S1–S6), with the connecting region between S5 and S6 forming the Nav1.5 channel pore (pore), exhibiting voltage-gated properties, and determining channel permeability and selectivity.^[82–84]^ Numerous mutations in the SCN5A gene causing LQT3 have been reported, with over 500 documented to date.^[85]^ Mutations at different sites in the SCN5A gene lead to varying degrees of impaired inactivation of slow Nav1.5 channels, generating sustained inward sodium currents during repolarization. This disrupts the balance of ions during the action potential plateau, causing prolonged action potential duration and QT intervals, which can lead to EADs and trigger fatal arrhythmias.^[86–88]^ Additionally, different SCN5A gene mutations may result in other phenotypes of hereditary arrhythmia syndromes or overlapping phenotypes.^[89]^ Therefore, in-depth research on the function and characteristics of the SCN5A gene may provide a theoretical basis for clinical diagnosis and preventive treatment.

LQT2 accounts for approximately 25% to 30% of LQTS cases, and the KCNH2 gene is the pathogenic gene for LQT2,^[90]^ also known as the hERG. It encodes the alpha subunit of the voltage-gated potassium channel (Kv11.1). Kv11.1 forms a complex with 4 alpha subunits and beta subunits encoded by KCNE2, mediating the rapid delayed rectifier potassium current (IKr) and participating in the repolarization of myocardial cell membranes.^[77,91]^ A recent single-center study of LQTS in the Chinese population revealed LQT2 to be the most common genotype. Over 700 pathogenic mutations have been identified in the KCNH2 gene, including missense mutations, nonsense mutations, splice mutations, frameshift mutations, and fragment deletions.^[92]^ Functional impairment of the hERG protein due to KCNH2 gene mutations can be categorized into 4 main mechanisms: reduced hERG protein synthesis, impaired channel protein transport,^[48,50]^ altered gating properties, and decreased potassium ion permeability. Most missense mutations result in impaired hERG channel protein transport,^[93]^ representing the most common mechanism for the loss of hERG protein function.^[94,95]^ LQT2 electrocardiographic manifestations include asymmetrical, low-amplitude, bifid, or notched T-waves in multiple leads. Potential triggering factors for arrhythmias associated with KCNH2 gene mutations include sudden noises, shocks, and emotional stress. Sudden auditory stimuli may induce the sudden release of local catecholamines, triggering arrhythmias. The regulation of Ikr current involves the activation of both β1 adrenergic receptors mediated by the AC/cAMP/PKA signaling pathway and α1 adrenergic receptors mediated by the PLC/DG/PKC signaling pathway.^[96,97]^

As early as the 1990s, the hERG gene was discovered in the original hippocampal cDNA library. In recent years, it has been found to be distributed in various locations, including hippocampal astrocytes and the cerebral cortex, regulating the excitability of the central nervous system (CNS).^[98,99]^ Ion movement across membranes is related to channel function, and proteins are the majority of neurotransmitters that regulate channels. Their metabolism often occurs with DNA as a template. Therefore, abnormal channel function and regulation may be the result of abnormal gene expression.^[100]^ Research has also confirmed the existence of epilepsy genes or candidate gene mutations in some human idiopathic epilepsies. Johnson et al study results suggest that if LQT2 patients are more prone to epilepsy, then the HERG gene may represent a novel epilepsy-related pathogenic gene.^[58]^

### 3.2. Mechanisms of epilepsy

Epilepsy is a common neurological disorder, with over 50 million epilepsy patients worldwide.^[101]^ According to the World Health Organization, the prevalence of epilepsy is 5.0‰ in developed countries and 7.2‰ in developing countries. In China, the prevalence ranges from 3.6‰ to 7.0‰, with higher incidence among children and adolescents, where over 50% of individuals experience their 1st seizure before the age of 20.^[102–106]^ However, despite reasonable treatment with antiepileptic drugs (AEDs) and other therapeutic approaches, only 70% to 80% of patients achieve effective seizure control. Approximately 20% to 25% of patients, despite receiving standardized monotherapy or combination therapy, still experience suboptimal control, leading to the classification of drug-resistant epilepsy.^[107–109]^

The brain’s information transmission and processing rely on the high-speed collaboration of neural networks between different parts. Normal network operation guides the precise and appropriate behavior of the organism. If there is an abnormality in a component or loop of the network, pathological changes quickly occur, often resulting in epileptic seizures. Epileptic seizures are now considered a consequence of abnormal excitability in neural networks, characterized by highly synchronized abnormal discharges of neurons. Gamma-aminobutyric acid (GABA) and glutamate are the most important inhibitory and excitatory neurotransmitters in the nervous system, and alterations in the GABA and glutamate systems are crucial mechanisms underlying epileptic seizures.^[110,111]^

Neurogenesis refers to the process of generating new neurons from neural precursor cells in specific brain regions. In the CNS of adult mammals, 2 regions capable of neurogenesis exist: the subgranular zone of the dentate gyrus in the hippocampus and the subventricular zone around the lateral ventricles. Neural stem cells or neural progenitor cells in these regions can actively undergo mitosis, demonstrating self-renewal potential, and can differentiate into neurons, astrocytes, or oligodendrocytes.^[112]^ Various harmful stimuli, such as epilepsy, ischemia, and hypoxia, can impact neurogenesis in these 2 regions. Under pathological conditions, neural stem cells in the subventricular zone and the dentate gyrus of the hippocampus in adult rats can proliferate and migrate toward brain lesions. However, this proliferation is transient and does not contribute significantly to therapeutic repair.^[113–115]^

In normal circumstances, neurons maintain stable neural activity by balancing excitatory and inhibitory signals.^[116]^ In epilepsy, certain factors lead to neuronal overexcitation. This may result from mutations in ion channels, imbalance of neurotransmitters, changes in postsynaptic membranes, and other factors. Neuronal electrical activity relies on the switching of ion channels. Mutations can disrupt ion channels, affecting the regulation of neuronal membrane potential and causing increased excitability.^[117]^ For example, mutations in sodium and calcium channels in some types of epilepsy may lead to abnormal neuronal excitation. Neurotransmitters are chemical signals that facilitate information transfer between neurons. Imbalances in neurotransmitter levels can destabilize the interaction between excitatory and inhibitory signals.^[118–120]^ GABA is a major inhibitory neurotransmitter, and in some epilepsy cases, impaired GABA function makes neurons more prone to excitation. The postsynaptic membrane is the junction between neurons, and structural and functional changes in the postsynaptic membrane may occur in epilepsy.^[121,122]^ These changes may include remodeling of the postsynaptic membrane, depolarization, and rapid repolarization after depolarization, all of which can lead to abnormal neuronal discharges.^[123,124]^ Epilepsy involves not only abnormalities in individual neurons but also dysregulation of entire neuronal networks. Network abnormalities can lead to the spread of discharges, affecting large brain regions and triggering generalized epileptic seizures.^[125–127]^ Genetic factors play a crucial role in certain types of epilepsy, while environmental factors such as head injuries, infections, and drugs can trigger or increase the risk of epilepsy.^[128]^

Considering these factors, the pathogenesis of epilepsy involves neuronal hyperexcitability, imbalance of ion channels and neurotransmitters, changes in the postsynaptic membrane, and abnormalities in neuronal networks. Different types of epilepsy may have distinct mechanisms, and ongoing research continues to deepen our understanding of this field.

### 3.3. Relationship between LQTS and epilepsy

LQTS and epilepsy are 2 distinct disorders, but research suggests that they may share some associations or common pathogenic mechanisms.

Genetic mutations may simultaneously affect ion channels in both the heart and brain neurons. For instance, mutations in certain ion channel genes may not only lead to prolonged QT intervals in the heart (LQTS) but also cause abnormal discharges in neurons, thereby increasing the risk of epilepsy.^[129]^ The gene specific to LQT1, KCNQ1, encodes the I_Ks channel in the heart and is also expressed in neurons. Mutations in this gene may result in ion channel dysfunction in both heart cells and neurons, increasing the likelihood of co-occurrence of LQTS and epilepsy.^[130]^ Epilepsy is a chronic, recurrent, and sudden-onset dysfunction of the CNS, characterized by abrupt high-frequency discharges of neurons in the cerebral cortex lesions and their spread to surrounding areas. Ion channels form the basis for the regulation of excitability in excitable tissues within the body. Therefore, it is currently believed that many cases of human idiopathic epilepsy are associated with mutations in genes encoding ion channel proteins, representing a type of ion channelopathy where abnormal firing of neurons is linked to mutations affecting sodium, calcium, and potassium ion channels. In summary, the relationship between LQTS and epilepsy involves shared genetic mutations affecting ion channels, potentially contributing to the co-occurrence of these 2 neurological disorders.

Gao et al^[16]^ provided a comprehensive review of research advancements in the precision diagnosis and treatment of potassium channel-related genetic epilepsy. Potassium ion channels play a crucial role in the electrical activity of neurons and directly participate in the mechanisms underlying epilepsy. Genes implicated in this context include KCNA1, KCNA2, KCNB1, KCNC1, KCND2, KCNQ2, KCNQ3, KCNMA1, KCNT1, among others. Several potassium channel genes and candidate drugs have been identified. Immune system involvement in epilepsy has garnered widespread attention in recent years, considering both innate and adaptive immunity can be activated due to CNS damage, leading to epileptic seizures. Chen et al^[14]^ elaborated on the intricate interaction between ion channels and the mTOR pathway. They highlighted the complexity of the interaction between ion channels (which play a distinct role in epilepsy occurrence and development) and the immune system. Skinner et al^[26]^ analyzed ion channel diseases contributing to sudden cardiac death, including LQTS, Brugada syndrome, and catecholaminergic polymorphic ventricular tachycardia. Cell action potentials driving the cardiac cycle are mediated by a series of specific depolarizing and repolarizing ion currents facilitated by ion channels. Changes in any of these currents or alterations in the availability of intracellular free calcium can make the myocardium prone to polymorphic ventricular tachycardia or ventricular fibrillation.

Several studies indicate a significant correlation between ion channels acting on LQTS and the nervous system. For instance, the Kv7.1 channel, which is implicated in LQT1 due to mutations in the KCNQ1 gene, not only exists in cardiac cells but also in neurons. The Kv7.1 channel’s primary role in neurons is to regulate the cell’s membrane potential, affecting the transmission of neural signals. Mutations in the KCNQ1 gene may lead to a reduction in the number of Kv7.1 channels, functional abnormalities, or abnormal cell membrane localization. These changes may not only impact cardiac cells but may also cause abnormalities in neurons, increasing the risk of abnormal neuronal discharges, leading to delayed repolarization, prolonged action potential duration, QT interval extension, and EAD. In neurons, EAD and abnormal discharges are associated with the occurrence of epilepsy. Gozalo et al^[131]^ induced epilepsy in patients with LQTS caused by mutations in the KCNQ1 gene.

KCNH2 is primarily expressed in the heart but, in certain situations, has been found to exhibit low-level expression in neural tissues, potentially involving the stability and excitability of neuronal membranes.^[132]^ Voltage-gated potassium channels, along with other ion channels, collectively regulate the membrane potential of neurons. Abnormal ion channel function may lead to changes in neuronal excitability, influencing the occurrence of epilepsy, as previously discussed. The level of ion channel function may be related to extracellular neurotransmitter levels, and some neurotransmitters are associated with epilepsy. Zamorano-León^[43]^ reported a case of a young woman experiencing frequent seizures and syncope during walking and at rest. Genetic testing revealed a de novo mutation (2587T→C) in exon 10 of the KCNH2 gene associated with LQT2. This mutation resulted in a premature termination codon substitution (R863X), leading to a deletion of 296 amino acids in the HERG channel. Changes in R863X in the HERG channel are likely associated with prolonged QTc intervals and epilepsy. This suggests that alterations in R863X in the potassium channel encoded by KCNH2 may predispose individuals to both epilepsy and LQT2 arrhythmias.

Nav1.5 channels are sodium ion channels on the membrane of cardiac muscle cells responsible for regulating the rise and fall of action potentials. While these channels are primarily found on cardiac muscle cells, some ion channels may also be expressed in neurons, playing a role in the regulation of neuronal excitability. Remme et al^[56]^ investigated the relationship between SCN5A gene mutations and arrhythmias, myocardial diseases, and epilepsy. Diseases related to acquired disorders of sodium channel function (myocardial ischemia, heart failure) and hereditary diseases secondary to mutations in the SCN5A gene encoding the cardiac sodium channel Nav1.5 are associated with life-threatening arrhythmias. SCN5A and Nav1.5 are expressed in cell types outside of cardiac muscle cells and various extracardiac tissues, and their functional roles in epilepsy, gastrointestinal motility, cancer, and innate immune responses are gaining more research attention. Additionally, recent research^[133]^ discovered that among 951 identified variants, 437 sodium channel variants met inclusion criteria. Of these, 141 variants were associated with epilepsy (SCN1/2/3/8A), 79 with neuromuscular phenotypes (SCN4/9/10/11A), and 149 with cardiac phenotypes (SCN5/10A). Sixty-eight variants were considered benign, as they were included in electrophysiologically characterized mammalian cell whole-cell patch clamp recordings of missense variants. A total of 38 pairs of missense variants were detected in different sodium channel genes, with 35 pairs resulting in similar functional consequences, indicating a high biophysical concordance of 92% between corresponding sodium channel variants (odds ratio = 11.3; 95% confidence interval = 2.8 to 66.9; *P* < .001).

Both LQTS and epilepsy involve electrophysiological abnormalities, specifically neuronal hyperexcitability and instability of electrical activity. While these abnormalities may occur in different tissues (cardiac and brain neurons), sharing certain electrophysiological features could lead to some overlap between the 2 diseases. Some drugs used to treat LQTS and epilepsy may affect both conditions. For example, certain AEDs may influence cardiac ion channels, affecting the QT interval and potentially triggering LQTS. Some genetic and environmental factors may simultaneously impact the risk of both LQTS and epilepsy, including gene mutations, lifestyle, and environmental exposure.

In summary, the connection between LQTS and epilepsy primarily lies in the potential sharing of some underlying electrophysiological abnormalities in the nervous system, and certain ion channel abnormalities may play a crucial role in both diseases. However, further in-depth research is still needed to gain a clearer understanding of the exact relationship between these 2.

## 4. Misdiagnosis reasons for LQTS and epilepsy

LQTS and epilepsy may present with symptoms such as syncope, seizures, and loss of consciousness, making it challenging for doctors to differentiate between the 2 during initial assessments.^[39]^ Both conditions can manifest as episodic symptoms, like syncope, and in certain situations, these episodes may appear quite similar, leading to potential misinterpretation by healthcare professionals. While LQTS is typically associated with electrophysiological abnormalities in the heart, and epilepsy involves abnormal electrical activity in brain neurons, in some cases, both conditions may coexist, further complicating the diagnostic process.

The electrocardiogram (ECG) of individuals with LQTS may show prolonged QT intervals, but not all LQTS patients exhibit obvious ECG abnormalities. Conversely, some epilepsy patients may have normal ECG results, adding complexity to the reliance on ECG for differential diagnosis.^[55,134]^ Accurately calculating the QT interval can be challenging, and even when the ECG is considered a necessary examination for differentiating between syncope and seizures, its interpretation is often not comprehensive. The QT interval is the time from the onset of the QRS complex to the end of the T-wave returning to the isoelectric line. Past studies have found that <40% of non-cardiology specialists and <50% of cardiology specialists can correctly calculate the QTc interval.^[135]^ Although ECG machines automatically provide calculated QT interval results, clinical practitioners are still required to have the ability to compute the QT interval.^[136]^ QT interval calculation on the ECG typically chooses lead II, and the calculation of the QT interval and RR interval takes the average of at least three consecutive heartbeats. Prolonged QT intervals (QTc of approximately 700 ms) are visible in multiple ECG leads, with lead II being particularly clear.

In addition to the limitations of the electrocardiogram for the diagnosis of LQTS and the EEG for epilepsy, their specificity and sensitivity are insufficient to definitively diagnose the diseases. The diagnosis of epilepsy relies more on the patient’s clinical presentation, and a normal EEG cannot entirely rule out the diagnosis of epilepsy, while an abnormal EEG cannot definitively confirm the presence of epilepsy. Reports indicate that 10% to 40% of epilepsy patients do not show abnormal discharges in multiple EEG examinations.^[137]^ Similarly, for LQTS, abnormal QT intervals cannot entirely confirm or rule out the diagnosis, and normal QT intervals cannot entirely rule out LQTS. Assessing the diagnosis of LQTS requires a combination of patient clinical symptoms, family history, exercise tests, catecholamine stress tests, and genetic diagnosis, among other information. Moreover, simple genetic mutation testing cannot predict clinical outcomes. Many gene carriers may not exhibit symptoms, and even individuals with the same gene mutation may present varying degrees of the disease.^[138]^ Therefore, in the same family, even with identical gene mutations, different disease manifestations may occur.

Ultimately, a comprehensive diagnosis requires considering multiple aspects of information, including clinical symptoms, family history, auxiliary examinations (such as ECG and EEG), as well as special tests and genetic diagnosis. This integrative diagnostic approach is more accurate in determining whether a patient has LQTS or epilepsy (Table 1).

**Table 1:** The points of clinical differentiation between epilepsy and LQTS.

Clinical manifestation	Epilepsy	LQTS
Aura	Symptoms of winter lobe origin may appear, such as vision, hearing, smell, digestive discomfort, somatosensory or emotional disturbances.	There is generally no aura, and occasionally palpitations occur when discomfort is greater than stress.
Ictal manifestation	Focal: abnormal sensations in a certain part of the body, such as acupuncture, warmth, electric shock or limb loss, auditory hallucinations, vision, smell attacks, taste attacks and vertigo attacks, muscle twitching in a certain part of the body. Generality: loss of consciousness accompanied by generalized compulsive clonic seizures or atonic seizures.	Sudden loss of consciousness accompanied by tonic-clonus or decreased muscle tone.
Tongue bite	Common	Infrequent
Eyes gaze	Common	Infrequent
Gatism	Common	Infrequent
Postictal manifestation	Dizziness, headaches, confusion, muscle aches, fatigue, and even numbness (Todd numbness).	After the attack, the heart rate quickly returned to normal, without special discomfort.
Family history	Probably exist.	Probably exist.
Birth history	There may be intrauterine hypoxia, birth injury, neonatal room rest, etc	There are generally no special circumstances.
Developmental history	May have childhood brain trauma, febrile convulsion, encephalitis history, etc	There are generally no special circumstances.
QT interval changes in ECG during exercise test	No significant change.	Ventricular repolarization and abnormal T-wave response.

ECG = electrocardiogram; LQTS = long QT syndrome.

## 5. Accurate diagnosis of LQTS and epilepsy

Both epilepsy and LQTS have the potential to lead to fatal outcomes. The annual incidence of untreated sudden cardiac death is in the range of 0.33% to 0.90%, with a syncope occurrence rate of 5.00%.^[139]^ Poorly controlled epilepsy patients may experience sudden death due to seizures. Once these 2 conditions are accurately diagnosed, patients and their families undergo significant physical and emotional distress. On one hand, this is due to the discomfort and harm symptoms may cause, and on the other hand, the fear of facing death at any moment. Emotional and psychological disorders such as anxiety and depression may also accompany these conditions in some patients.^[138,140]^ Therefore, precise diagnosis, standardized treatment, and psychological guidance are crucial for both epilepsy and LQTS patients. Below are suggestions for differential diagnosis from various aspects, including prodromes of clinical episodes, medical history, family history, developmental history, and auxiliary examinations.

Despite proposing a diagnostic approach to differentiate between epilepsy and LQTS, it is acknowledged that the correct diagnosis of these 2 conditions remains challenging. Currently, the diagnosis of LQTS heavily relies on genetic testing. However, genotype variations may not exclusively impact cardiac manifestations. For instance, mutations associated with LQT2 can predict both arrhythmias and epilepsy, with LQT2 and epilepsy being independent risk factors for each other.^[141]^ Therefore, clinicians should maintain a proper diagnostic perspective. When patients experience unexplained recurrent syncope or seizure-like episodes, comprehensive 24-hour ambulatory electrocardiography and awake-sleep electroencephalography should be conducted whenever possible, along with the recording of long-term electrocardiography and electroencephalography if conditions permit. For all cases of generalized seizure episodes, a comprehensive electrocardiogram screening should be performed. For LQTS patients, especially LQT2, who present with recurrent unexplained loss of consciousness or seizure-like episodes, long-term awake and sleep-deprived electroencephalography examinations should be conducted. Regardless of whether the patient has LQTS, relevant epilepsy examinations should also be performed.

Reports have indicated that epilepsy patients misdiagnosed as LQTS failed to record arrhythmias during the implantation of an implantable cardioverter-defibrillator (ICD) and eventually had to remove the ICD.^[142]^ Such misdiagnoses and treatments not only cause substantial harm to patients but may also damage the doctor–patient relationship, leading to patient distrust. Therefore, while increasing awareness of these 2 conditions, clinicians also need to clearly explain the complexity of the diagnosis and encourage patients and their families to provide more detailed information about the history of episodes or past medical history. This collaborative effort helps both healthcare providers and patients work together towards better disease management.

## 6. Treatment of LQTS and epilepsy

The primary emphasis in treating both diseases is lifestyle modification. Patients should avoid vigorous exercise, including water-related activities like swimming, and stay away from noisy environments. Guidelines recommend patients engage in mild exercises such as billiards, bowling, and golf.^[143,144]^

The treatment of LQTS mainly involves using high-dose beta-blockers, ICD for tachycardia suppression, and potentially sympathectomy, especially for high-risk patients.^[145]^ In the treatment of epilepsy, AEDs constitute the main therapeutic approach, with two-thirds of patients achieving seizure control through medication.^[146,147]^ However, drug therapy does not significantly improve long-term prognosis. For some refractory focal epilepsy patients, surgical resection of the lesion is an effective method for achieving long-term seizure control, although its application is currently limited due to eligibility constraints.^[109,148,149]^ As there are similarities in the pathophysiological mechanisms of both diseases, treatment measures may produce adverse reactions in patients, particularly impacting the other condition.

In addition, we need to pay attention to the factors that affect ventricular repolarization, including screening for medications that may prolong QT interval, such as certain antiarrhythmics, macrolide and fluoroquinolone antibiotics, antipsychotics, methadone, and varenicline, as these drugs can potentiate proarrhythmic risk. At the same time, electrolyte abnormalities, particularly hypokalemia, hypomagnesemia, and hypocalcemia, are well-recognized contributors to QT prolongation and should be carefully monitored.^[150,151]^ Therefore, caution is necessary when devising treatment plans, tailoring them based on individual patient conditions, avoiding drugs that may prolong the QT interval, and maintaining proper electrolyte balance, adequate hydration, and minimizing stimulating factors.^[71,150–152]^ Failure to correct a misdiagnosis in a timely manner may impose financial burdens and health hazards on patients. While providing relevant treatment recommendations, close monitoring of drug efficacy and adverse reactions is essential.

The impact of phenobarbital is more pronounced in some studies.^[153]^ Recent research indicates that LQT2 patients taking AEDs have a higher probability of experiencing cardiac events. Especially for patients with prolonged QT intervals, the risk of cardiac events is higher, particularly when using sodium channel blockers. However, this risk improves with concomitant use of beta-blockers.^[154,155]^ Female LQTS patients are at a higher risk of developing arrhythmias, and the incidence of LQTS and drug-related arrhythmias is also higher in females. Therefore, when choosing AEDs, factors such as the subtype of LQTS, the mechanism of action of AEDs, whether beta-blockers are used in conjunction, and gender need to be considered for patients with prolonged QT intervals.^[149,156]^ However, literature results on this subject from basic and clinical research are contradictory. Some studies suggest that lamotrigine, phenytoin, and phenobarbital can inhibit crucial potassium channels in myocardial cells, slowing depolarization and contraction of myocardial cells and tissues.^[157]^ Yet, in clinical studies of healthy individuals and newly diagnosed elderly epilepsy patients, lamotrigine does not alter the QT interval or QRS duration.^[158]^ Sodium phenytoin is believed to be associated with QT interval prolongation and arrhythmias caused by atrioventricular conduction block.^[159–161]^ However, some research reports no effect of sodium phenytoin on arrhythmias and even a potential reduction in the risk of arrhythmias.^[162]^ Currently, there is a wealth of reports on the impact of drugs and treatments on epilepsy and the QT interval. For example, the treatment of postictal depression, serotonin reuptake inhibitors, and tricyclic antidepressants may increase the QT interval, while the effect of sertindole is more pronounced.^[163,164]^ The ketogenic diet is considered not to increase the QT interval.^[165]^ The AMPA receptor antagonist perampanel also does not increase the QT interval.^[165]^ However, excessive use of methadone and opioids may increase the QT interval.^[166]^

Currently, there is a lack of in-depth research on the impact of AEDs on the QT interval in LQTS patients. Although some drugs may not have a significant effect on the QT interval in normal patients, the genetic coding and channel function of LQTS patients have already changed, making the situation potentially different. However, conducting such trials is challenging due to practical considerations and ethical factors. AEDs are not only used to control seizures but are also widely used to treat conditions such as mood stabilization and neuropathic pain. Therefore, when using AEDs for treatment, close attention should be paid to the patient’s clinical condition, and changes in the QT interval on the electrocardiogram should be carefully monitored. In cases where either diagnosis is not fully met, clinicians should always maintain awareness and sensitivity to both diseases. Paying particular attention to the effectiveness of the treatment plan and adverse reactions and making timely adjustments is crucial. If a patient shows no signs of relief under standard treatment for an extended period, consideration should be given to the possibility of misdiagnosis and potential complications.

## 7. Conclusion and future perspective

Epilepsy and LQTS share certain similarities in clinical presentation and pathophysiological mechanisms, particularly in the case of LQT2. Due to these similarities, differentiation and definitive diagnosis become challenging, primarily due to similarities in clinical presentation, insufficient sensitivity and specificity of auxiliary examinations, and potential inadequacies in the thought process of diagnosing physicians. A careful understanding of the clinical characteristics of cardiac-origin seizures, similarities and differences in medical history, comprehensive long-term electrocardiogram and EEG examinations, along with specific genetic testing and exercise stress testing, can aid in ensuring accurate differentiation and definitive diagnosis. Given the significant clinical risks associated with both diseases, timely and definitive diagnosis is crucial for developing effective treatment plans and conducting disease education, contributing to a reduction in mortality rates. When dealing with patients exhibiting prolonged QT intervals, physicians should exercise caution in the selection of AEDs, avoiding medications that may increase the QT interval or induce arrhythmias.

Clinicians should closely monitor patients’ clinical manifestations, response to treatment, and adverse reactions, especially when using AEDs in patients with prolonged QT intervals. When inquiring about the patient’s seizure history and medical history, physicians should carefully consider possibilities related to both the heart and brain. Additionally, monitoring patients’ condition after medication and adjusting drug doses and treatment plans promptly are essential. Active participation and cooperation from patients and their families are crucial factors for successful treatment. Cultivating good compliance during treatment enables better collaboration, ensures patients adhere to lifestyle changes as per medical advice, and promptly report any discomfort or abnormal conditions.

In conclusion, the importance of accurate diagnosis and differentiation between epilepsy and LQTS cannot be overstated. Through comprehensive clinical assessments, auxiliary examinations, and specialized medical judgment, physicians can better formulate treatment plans, improve patients’ quality of life, reduce psychological burdens on patients and their families, ultimately leading to better treatment outcomes.

## Author contributions

**Writing – original draft:** Qing Li, Ling-Bing Meng.

**Writing – review & editing:** Qing Li, Ling-Bing Meng.
